# Effect of Lexical-Semantic Cues during Real-Time Sentence Processing in Aphasia

**DOI:** 10.3390/brainsci12030312

**Published:** 2022-02-25

**Authors:** Niloofar Akhavan, Christina Sen, Carolyn Baker, Noelle Abbott, Michelle Gravier, Tracy Love

**Affiliations:** 1 School of Speech, Language, and Hearing Sciences, San Diego State University, San Diego, CA 92182, USA; csen7960@sdsu.edu (C.S.); cjbaker@ucsd.edu (C.B.); ntodd6857@sdsu.edu (N.A.); tlove@sdsu.edu (T.L.); 2 Department of Cognitive Science, University of California San Diego, San Diego, CA 92122, USA; 3 Joint Doctoral Program in Language and Communicative Disorders, San Diego State University/University of California San Diego, San Diego, CA 92182, USA; 4 Department of Speech, Language and Hearing Sciences, California State University East Bay, Hayward, CA 94542, USA; michelle.gravier@csueastbay.edu

**Keywords:** semantic cue, eye tracking, real-time sentence processing, syntax, aphasia

## Abstract

Using a visual world eye-tracking paradigm, we investigated the real-time auditory sentence processing of neurologically unimpaired listeners and individuals with aphasia. We examined whether lexical-semantic cues provided as adjectives of a target noun modulate the encoding and retrieval dynamics of a noun phrase during the processing of complex, non-canonical sentences. We hypothesized that the real-time processing pattern of sentences containing a semantically biased lexical cue (e.g., the venomous snake) would be different than sentences containing unbiased adjectives (e.g., the voracious snake). More specifically, we predicted that the presence of a biased lexical cue would facilitate (1) lexical encoding (i.e., boosted lexical access) of the target noun, snake, and (2) on-time syntactic retrieval or dependency linking (i.e., increasing the probability of on-time lexical retrieval at post-verb gap site) for both groups. For unimpaired listeners, results revealed a difference in the time course of gaze trajectories to the target noun (snake) during lexical encoding and syntactic retrieval in the biased compared to the unbiased condition. In contrast, for the aphasia group, the presence of biased adjectives did not affect the time course of processing the target noun. Yet, at the post-verb gap site, the presence of a semantically biased adjective influenced syntactic re-activation. Our results extend the cue-based parsing model by offering new and valuable insights into the processes underlying sentence comprehension of individuals with aphasia.

## 1. Introduction

One property of language processing is the ability to integrate sentential constituents and establish linguistic relationships between non-adjacent pieces of information. This latter process creates syntactic dependencies and is critical for the determination of the underlying meaning of the sentence. To successfully understand an utterance, the listener must assign appropriate roles of the nouns to the linked verbs in the sentence. This is accomplished automatically (i.e., it is an automatic process which is a reflexive, unconscious, moment by moment operations that unfolds in real-time during sentence processing) through thematic role assignment (for example, determining which noun is the agent or actor and which noun is the theme or object of the verb). In English (which has a strict subject–verb–object word order), this process aligns quite nicely with the order of input of a simple active sentence (1a); that is, the first noun encountered is the actor or agent, and the noun after the verb is the object. This process is also simple for more complex sentence constructions that maintain canonical word order, such as those found in subject-relative constructions (1b):

(1a) The girl *hits* the boy.
(1b) The girl that *hits* the boy is angry.
(1c) The boy_i_ that the girl *hit*_i_ <the boy> was angry.

This automatic process of assigning thematic roles becomes more challenging for listeners when the sentence structure deviates from the canonical word order. Sentence (1c), above, is an example of a non-canonical sentence (object-relative construction). In this example, the object of the verb ‘hit’ (/the boy/) is fronted or displaced to the beginning of the sentence, causing it to be structurally separated from its underlying (post-verb) position. When processing this sentence, the listener, upon hearing the verb, must link the verb to its object (noted by the subscript ‘i’). This retrieval process allows for its integration with the syntactic and semantic properties of the verb to facilitate interpretation. 

Numerous studies have found evidence of re-activation of the direct object at the gap site (the position from where the noun phrase has been displaced is known as a gap; the ‘i’ indexation represents a link between the verb and its structurally licensed direct object) using various online methodological approaches including probe recognition tasks [1], cross-modal priming tasks [2], and eye-tracking [3,4]. Although dependency linking occurs rapidly and relatively automatically in neurologically healthy individuals, the associated processing cost is higher in non-canonical sentences compared to canonically ordered constructions due to the need of forming long-distance dependencies [5,6,7,8,9]. Theoretical models of sentence processing, namely cue-based parsing, make specific predictions regarding the processing costs associated with long-distance dependencies. According to these models, the success (as measured by reaction time methods) of retrieving the displaced constituent (i.e., syntactic dependency linking) is a function of the degree of interference from similar items in memory that compete with the retrieval of the target item [9,10,11]. The higher the interference, the more likely the wrong target will be retrieved. Although these models are based on data from neurotypical adults, interference has also been found to contribute to comprehension impairments in post-stroke aphasia [3,12,13]. For individuals with aphasia, the presence of interference can overwhelm the impaired system and lead to breakdowns in comprehension [14,15,16,17], thus the focus of the current study (see Section 1.3 below).

It is generally accepted that comprehension deficits in aphasia are not the result of an irrevocable loss of stored linguistic representations [15,18], but rather stem from disruptions to the automatic operations involved in sentence processing. According to some researchers, these deficits stem from processing impairments at the lexical level [16,19,20,21,22,23] which includes disruptions to the processes of lexical access and integration. These are fundamental mechanisms that provide the system with timely lexical information and allow for the incorporation of that information into a syntactic frame. Lexical-level deficits can emerge from impairments of representational encoding and retrieval which can amplify the effects of interference. As described below, the current paper investigates whether semantic-level manipulations during encoding can reduce interference effects and alleviate retrieval difficulties during sentence processing [24,25]. We expect the semantic-level manipulations during encoding to facilitate (1) lexical processing (i.e., boost representational access) and (2) online syntactic dependency linking (i.e., reduce interference effect) in neurologically unimpaired adults as well as in individuals with aphasia.

### 1.1. Interference Effect during Sentence Processing

As discussed previously, under many theoretical accounts, the successful linking processing of the verb “chased” in object-relative sentences such as (2) depends on the retrieval of its syntactic direct object *bear* upon encountering the verb *chased* [26,27,28].

(2) It was the bear_i_ that the hunters chased_i_ in the cold forest yesterday.

According to the cue-based parsing retrieval theory, a memory representation of the noun *bear*, is formed (encoded) as a bundle or vector of certain syntactic and semantic features such as [+nominative; +animate; +singular] that are activated when the noun is first encountered in the sentence. These features remain active in some form of memory—but outside the focus of attention—as the sentence constituents unfold. When the comprehender reaches a retrieval point (e.g., the verb), the representation of the noun phrase (NP; “the bear”) must be integrated into the structural frame to be assigned its thematic role. Cue-based parsing theory assumes that dependencies are resolved via a direct-access operation based on their representational content (i.e., content addressability) [29,30,31]. For example, at the verb ‘chased’ in sentence (2), a retrieval mechanism based on linguistic and contextual features is assumed to be immediately triggered, which seeks out a representation with the [+nominative; +animate] features (i.e., something that can be chased). During this content-addressable search, these features or retrieval cues are matched against all possible candidates (i.e., recently activated items) in memory. The likelihood of retrieving a given item is determined by the strength of the match between the features encoded with a given item and the features contained in the retrieval cue.

Although the cue-based parsing approach does not make specific predictions regarding the quality of encoding having an impact on retrieval probability and latency, recent studies have shown that enriching a word, via the addition of modifiers, facilitates its subsequent retrieval compared to conditions in which the same word is left unmodified [24,25,32,33,34]. In a self-paced reading study, Hofmeister (2011) investigated reading times in neurologically unimpaired participants for sentences which contained a critical noun that was either modified by zero, one, or two adjectives (low, mid, and high complexity conditions, respectively); see (3) below for an example of the high complexity condition. Note that the brackets, parentheses, and indexation have been added to highlight the manipulation but were not used in the study itself.

(3) It was [the injured and dangerous bear]_i_ that the hunters chased_i_ in the cold forest yesterday.

This study reported decreased reading times for the main verb for items in the highest complexity condition (i.e., where the direct object noun was preceded by two adjectives) compared to the other conditions. In a similar experiment, such findings were also observed for nouns that were semantically richer/more specific (e.g., “soldier”) compared to less semantically rich/less specific (e.g., “person”). Hofmeister (2011) interpreted these results as evidence that, for unimpaired comprehenders, the addition of semantic and syntactic features increased the uniqueness of the target representation compared to other lexical items in the sentence and facilitated retrieval of information and subsequent integration later in the sentence. This finding is in accordance with the predictions of encoding interference, which is assumed to arise from competition associated with the encoding of items with similar features [35]. By increasing the distinctness of the target item, a higher-quality representation was created, reducing encoding interference. An additional finding was increased encoding times for the more complex NP, which may have indexed additional cognitive effort required to perform combinatorial processing (i.e., incorporating the adjectives into the NP). Increased cognitive effort and the extended time dedicated to the NP within the auditory signal may have both served to raise the salience or activation level of the representational network [11,36].

### 1.2. Evidence from Aphasia of the Effect of Lexical Processing Deficits on Syntactic Processing 

As previously mentioned, lexical processing accounts suggest that auditory comprehension deficits in individuals with aphasia (IWA) are mainly due to lexical processing impairments. One such account is the delayed lexical activation hypothesis [16,19], which claims that slowed lexical activation precludes the timely formation of syntactic structure building as the parser is not provided with the necessary lexical information when needed. Delayed lexical activation occurs in both canonical and non-canonical sentences; however, when a delay occurs in non-canonical constructions, it feeds the syntactic processor too slowly, throwing off retrieval, which results in comprehension breakdowns. Evidence for this hypothesis comes from studies with IWA that have found delayed activation of NPs when they were first encountered in an auditory object-relative sentence as well as at the gap site following a verb [16]. The delayed re-activation of the displaced direct object NP was taken as evidence that IWA are able to perform syntactic computations; however, this process is slowed compared to neurologically unimpaired comprehenders. In this same study, Love and colleagues (2008) found that when the rate of speech input was slowed, IWA showed both on-time initial lexical activation and re-activation at the post-verb gap site, and critically, improved final sentence comprehension. Several other studies with IWA using various methods have also found either delayed lexical activation and/or delayed integration of lexical information into the sentence context [21,37]. For example, Swaab and colleagues (1997), in an event-related potential study, reported that IWA had an N400 component (a neurophysiological index of semantic integration processing) upon hearing a sentence-final word that violated the sentential-semantic constraints (e.g., “The girl dropped the candy on the sky”) that were either reduced in amplitude or delayed compared to neurologically unimpaired individuals [20,21]. Other studies using the eye-tracking-while-listening paradigm (ETL) have also indicated processing deficits in IWA. In an ETL visual world paradigm, participants are asked to listen to sentences over headphones while viewing a visual array displaying four items (characters mentioned in the sentence as well as item(s) unrelated to the sentence). The timing of eye gazes to the characters on the screen while listening to the unfolding sentences is argued to index underlying linguistic processing in real-time [38,39,40,41]. Many studies using ETL consistently indicated a late-emerging influence of competitor interpretations during sentence processing for IWA (i.e., interference effects) in incorrectly comprehended trials, providing further evidence for delayed integration [12,13,23]. In these ETL studies, the delay in lexical activation is proposed to result in interference effects when subsequent sentence constituents are activated during auditory processing. Altogether, these studies support the idea that encoding deficits at the lexical level plays a significant role in sentence processing, and consequently comprehension deficits for IWA. These findings are also in line with theoretical models of sentence processing such as the cue-based parsing theory which proposes that representational encoding at the word level is the core component of the sentence processing mechanism. 

### 1.3. The Current Study

**Figure 1:**
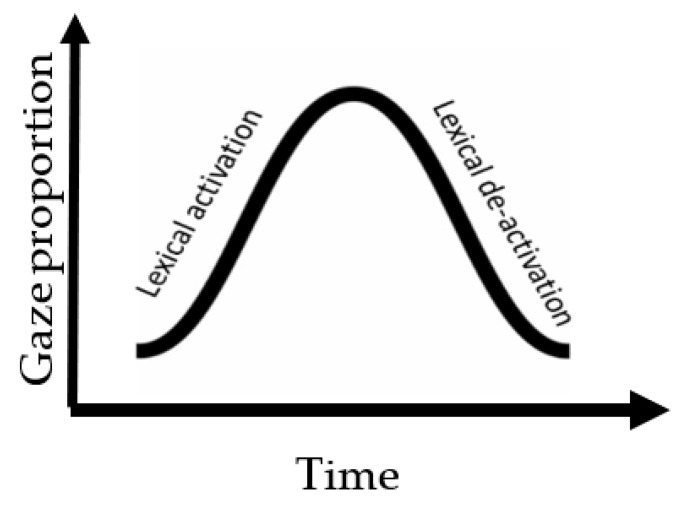
An illustration of the overall pattern of lexical processing in an ongoing sentence that involves an activation and de-activation phase. Activation is represented by increase in gaze proportion toward the heard item in the sentence while de-activation is represented by reduction in gaze proportion over time.

Using the eye-tracking-while-listening method, we investigate whether local contextual information at the semantic level (provided by an adjective preceding a target lexical item) can be used to facilitate the representational encoding of the nouns for listeners with and without aphasia during sentence processing. We further investigate whether the encoding pattern has any downstream effects on the retrieval of the target representations during dependency linking for both groups. Previous studies on this topic have primarily used self-paced reading paradigms to explore these effects during real-time sentence processing [24,25]. To tap into real-time auditory sentence processing, we employed the ETL method with a visual world paradigm (VWP). This method allows us to explore the time course of the proposed manipulation and its effect on processing throughout the sentence. As mentioned above, the way in which a word is encoded during sentence processing will impact the processing of subsequent constituents in the sentence. Therefore, to distill the encoding process of nouns, we examined their activation and de-activation patterns during an ongoing stream of the sentence (see Figure 1). The activation pattern is an indication of processing phonological and semantic features to access the target item (manifested as an increasing pattern of gaze movement toward an item). The de-activation pattern is an indication of a change in the level of representation at that time. Here, we operationalized de-activation as representing a shift **from** integrating the previously processed constituent into the sentence structure **to** accessing the new input. This is manifested as a decreasing pattern of gaze movement away from an item. Moreover, to distill the retrieval process that is involved in the verb-frame window, we examined the re-activation of the displaced object and its interference with distractor nouns in the sentence. The interference pattern is an indication of the competitive processing between the nouns that are lingering to be linked to the verb for the means of thematic role assignment. 

Table 1 demonstrates the different types of gaze movements that can be used as a metric to reflect varying stages of sentence processing including lexical and structural processing.

Here, we hypothesized that the lexical-semantic cues (in form of adjectives, see examples 4a and 4b) would facilitate (1) lexical encoding (i.e., boost representational access) and in turn (2) on-time syntactic retrieval or dependency linking (i.e., increasing the probability of on-time lexical retrieval at the gap site) during auditory sentence processing for both groups. Based on prior research, we anticipate that the overall pattern of online processing in individuals with aphasia to be delayed across conditions compared to the pattern in neurologically unimpaired individuals [13,16,23].

(4a) Unbiased adjective: The eagle saw the voracious snake_i_ that the bear cautiously encountered_i_ <the snake> underneath the narrow bridge.
(4b) Biased adjective: The eagle saw the venomous snake_i_ that the bear cautiously encountered_i_ <the snake> underneath the narrow bridge.

## 2. Methods

### 2.1. Participants 

Eleven individuals with chronic aphasia (IWA: female = 5, M_age_ = 54.2 years, SD_age_ = 8.2) and 11 age-matched controls (AMC: female = 7, M_age_ = 61.9, SD_age_ = 2.3) were recruited for this study. The inclusion criteria for both groups were as follows: participants were monolingual native English speakers with no exposure to a foreign language before the age of six; right handed (premorbidly for IWA); had no self-reported history of emotional or learning disorders or drug abuse and had normal to corrected self-reported vision and hearing. IWA had to have experienced a single left-hemisphere stroke at least 6 months prior to participation to control for the effect of spontaneous recovery. The diagnosis and severity of aphasia were assessed using standardized aphasia examinations, the Boston Diagnostic Aphasia Examination (BDAE-version 3; [42]) and the Western Aphasia Battery-Revised (WAB-R; [43]), and were confirmed by clinical consensus. Sentence comprehension ability was assessed using the S.O.A.P. Test of Sentence Comprehension [44] (see Table 2); IWA participants in this study demonstrated comprehension deficits, which we defined as at- or below-chance performance on the comprehension of sentences with non-canonical word order (object relatives and passives). The neurologically unimpaired age-matched participants additionally had no self-reported history of brain injury. Participants were excluded from this study if they did not meet the above criteria or were unable to understand directions and complete this study.

All participants were tested at the Language and Neuroscience Group Laboratory at San Diego State University and were paid $15 per session. A review of treatment history reveals that six of our seven participants had received prior treatment for sentence-level deficits, though the extent of treatment (number of sessions, type of treatment, and treatment response) was not available.

### 2.2. Materials 

This study utilized eye-tracking-while-listening with a visual world paradigm (ETL-VWP) to measure auditory sentence processing in real time. In this paradigm, participants listen to sentences over headphones while viewing a 2 × 2 visual array displaying four items (three characters mentioned in the sentence and one item unrelated to the sentence). The timing of eye gazes to the characters on the screen while listening to the unfolding sentences, such as those shown in Table 3, is argued to index underlying linguistic processing in real time [45,46].

#### 2.2.1. Visual Stimuli 

Visual stimuli consisted of freely available black-and-white line drawings of animals obtained from the internet and clip art resources that were resized to 450 × 450 pixels. During each trial, 4 images were displayed on the screen. Three of the images corresponded to each of the nouns in the experimental sentence. The fourth image was an unrelated control (e.g., “cat” in Table 3, above). The location of the images was counterbalanced across trials such that pictures corresponding to each noun in the sentence appeared equally as often in the 4 quadrants.

*Visual stimuli pretesting*: All images used in the experiment had 90% or greater name agreement on a naming pretest conducted with college-aged students naive to the goals of the present experiment (*n* = 34, M_age_ = 20.1 years, SD = 1.4).

#### 2.2.2. Sentence Stimuli 

The experimental sentences consisted of 30 sentence pairs (60 sentences total) containing non-canonical object-relative constructions that involved a long-distance dependency linking the displaced object (e.g., /snake/) and the relative clause verb (e.g., /encountered/; see Appendix A for the full list of sentences). These sentences were presented in two conditions: with a semantically neutral adjective preceding the displaced NP (i.e., the unbiased adjective condition), or with a semantically related adjective preceding the displaced NP (i.e., the biased adjective condition; see Table 3). The unbiased condition was included to control for the potential effect of the presence of a modifier increasing the salience of the NP (i.e., cognitive effort related to combinatorial processing) and to allow for isolation of the unique effect of the semantic information provided by the adjective (i.e., feature enrichment). In addition to these experimental sentences, 60 canonical sentence structures were included as non-experimental filler sentences. All sentences were recorded by a native English-speaking female at an average rate of 4.47 syllables per second. Each sentence trial was followed by a yes/no question to ensure that participants attended to the sentences. 

*Sentence stimuli pretesting*: Two pretests were conducted to ensure the selection of strong semantically related experimental adjective–noun pairs in the biased condition. In the first pretest, neurologically unimpaired college-aged participants (*n* = 34, M_age_ = 20.1 years, SD = 1.4) were shown a series of 120 black and white line drawings one at a time and were instructed to generate a descriptive word (an adjective) that corresponded with the image pictured. Sixty adjective–noun pairs were chosen for which a minimum agreement criterion (i.e., the concurrence of adjective choice (exact or semantically related) across participants) of 50% was met (M = 61%, SD = 10%). As a follow-up, a second pretest assessing semantic relatedness was conducted on the sixty adjective–noun pairs that were generated from the first pretest (e.g., “venomous snake”). A separate group of neurologically unimpaired college-aged participants (*n* = 23, M_age_ = 23.3 years, SD = 3.7) rated the semantic relatedness of each adjective–noun pair using a 5-point Likert scale (1 = Not Related; 5 = Highly Related). The thirty adjective–noun pairs with the highest ratings were selected for the ETL-VWP experiment (M = 4.59, SD = 0.41). To create an unbiased match for each of the experimental sentences, unbiased or neutral adjectives were chosen that were matched for syllable length, lexical frequency, and phonemic onset (*t*(59) = 0.08, *p* = 0.94).

### 2.3. Procedure 

**Figure 2:**
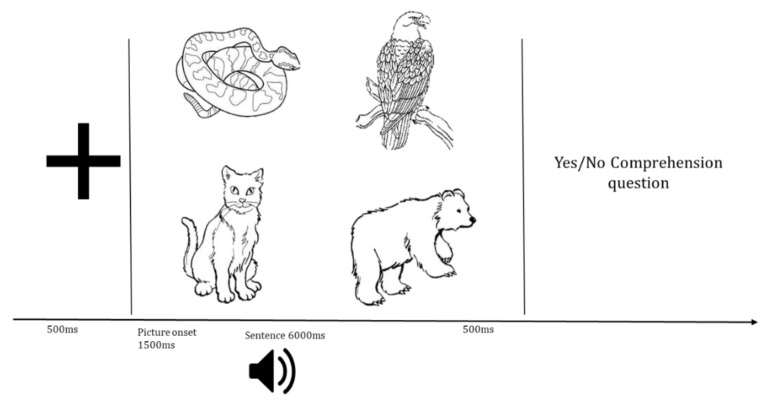
Example of a visual world eye-tracking paradigm. The speaker represents the auditory sentence.

This was a within-subjects experiment in which the trials were distributed and counterbalanced across 4 visits. Visits were spaced a minimum of one week apart. At the beginning of each visit, 10 practice trials were conducted to ensure understanding of the task. During the practice trials, the experimenter provided feedback as necessary. During each visit, participants were seated 60 cm from a computer screen with an attached Tobii X-120 eye-tracker and wore over-the-ear headphones for auditory stimulus presentation. The eye-tracker was calibrated at the beginning of each experimental session. Across each trial, gaze location was sampled at a rate of 60 Hz (every 17 ms) from both eyes. Stimuli were presented using E-Prime 2.0 software (Psychology Software Tools, Pittsburgh, PA, USA). Each trial began with a fixation cross presented for 500 ms, followed by a blank screen for 250 ms. Next, the four-picture display was presented for 1500 ms before the auditory sentence began and remained on screen for 500 ms after the sentence ended (see Figure 2). To ensure that all participants were attending to the sentences, following each trial, an offline measure was administered during which participants heard a question related to the sentence (e.g., was the bear under the narrow bridge?). Participants were instructed to respond as quickly as possible with a binary decision via a button box (YES/NO) using their left, non-dominant hand. The questions were either related to the action of the first or the third noun phrase of each sentence so as not to bring specific attention to the target displaced object NP. Half of the questions were designed to elicit a YES response.

### 2.4. Analysis Approach 

Preprocessing and analyses of eye-tracking data were performed using the eyetrackingR package [47] in R (R Core Team, 2019). In this study, gaze data from 60 trials across 22 individuals (11 in each AMC and IWA group) were sampled. All data across both groups and conditions were inspected to ensure that gaze patterns for the initial NP were evident. Furthermore, visual inspection revealed that for two sentences (in the biased condition) there were no discernable gazes to the first noun in the sentence (N1) after the auditory presentation of N1. Based on the rationale that lack of gazes to NP1 reflected either technical errors in gaze sampling during data collection or listeners’ difficulty distinguishing the visual items on the screen, data from these two sentences were removed from further analysis. Moreover, data from one sentence (from the unbiased condition) was excluded from analysis as the gaze patterns reflected semantic biasing towards a distractor noun in the sentence. In total, data from the 57 remaining experimental sentences were subjected to further analyses.

#### 2.4.1. Preprocessing 

Preprocessing of the eye-tracking data was conducted to check for trackloss, aggregate the data points across trials, and group them into temporal bins. Trackloss occurs when the gaze data are unavailable for both of the participant’s eyes (e.g., when they turn away or blink), which results in the validity of recorded gaze location being low (Tobii’s acceptable validity range is 0–2 on the scale of up to 4). Trials in which the trackloss proportion was greater than 25% were excluded from further analyses resulting in the removal of data from 19% of the trials. After reviewing the number of remaining trials available for analysis, it was determined that any participant who had more than 50% of their trials excluded due to the criteria listed above was to be removed from further analysis. This resulted in the exclusion of three participants (2 in the AMC and 1 in the IWA group) from the dataset. Data from the remaining 9 AMC and 10 IWA participants were aggregated across trials and aggregated into 100 ms time-bins. This approach is used as a strategy to account for the inherent dependency in time-series eye-tracking data which can inflate type I error rates. For each bin, the proportion of gaze within each AOI from the binary response variable (within or outside of an AOI) were estimated [48]. Gaze proportions were then subjected to statistical analysis (described below).

#### 2.4.2. Statistical Analysis Approaches 

Growth curve analysis (GCA) was used to explore the dynamic patterns of gaze movement over time in a preselected window of interest within the sentence. The GCA approach has been widely used in the analysis of gaze data in the visual world paradigm [49,50,51,52,53,54]. GCA is a multi-level modeling technique specifically designed to capture change over time using orthogonal polynomials [50]. The effects of the variables of interest on the polynomial terms provide a way to quantify and evaluate those effects on statistically independent (i.e., orthogonal) aspects of the gaze proportion trajectory. In the GCA approach, the level 1 model captures the overall gaze time course, with the intercept term reflecting the average overall gaze proportion. The linear term reflects a monotonic change in gaze proportion (similar to a linear regression of gaze proportion as a function of time) while the quadratic term reflects the symmetric rise and fall rate around a central inflection point [50]. The level 2 submodels capture the fixed effects of experimental conditions or group effects (categorical variables) on the level 1 time terms. The models in the current study included random effects of participants and items on intercept, linear, and quadratic time terms. Moreover, random slopes for condition were added per subject to achieve a maximal random effects structure [55]. Using the GCA approach, the fixed effects of variables of interest were added individually and their effects on the model were evaluated using model comparisons in order to examine whether a particular effect made a statistically significant contribution to model fit. Improvements in model fit were evaluated using −2 times the change in log-likelihood, which is distributed as *x*^2^ with degrees of freedom equal to the number of parameters added [48]. In this study, all analyses were conducted with the statistical software R-3.2.1, using the package LmerTest [56]. 

Cluster analysis was used to determine whether there were any time windows in which the looking patterns significantly differed between conditions (e.g., biased versus unbiased) within groups. The rationale of this method is to identify whether there is a series of consecutive time bins that show a significant effect of conditions. If the number of the consecutive time bins is larger than the observed null distribution, we can be confident that that the gaze pattern is different for the two conditions during the specified time window. Cluster analysis has been used in EEG studies [57] and in the visual world paradigm [52,58,59]. In this method, a separate test for the critical interaction at each individual time-bin was conducted (see below). If the time bins (20 ms) pass a determined threshold (*p*-value smaller than 0.05), then the adjacent time bins are clustered together. Finally, to correct for multiple comparisons, a non-parametric permutation test was conducted to determine the *p*-value for given cluster size. For this analysis, we used the eyetrackingR divergence analysis package [47].

## 3. Results 

*Offline processing*: Recall that participants were asked a yes/no question after each trial to ensure that they paid attention to the sentences. While these data were not used to inform the online analysis, we conducted a mixed-effects logistic regression model to explore group and condition differences. The results revealed an effect of group (AMC and IWA); specifically, the IWA group performed worse than the AMC group (estimate = −1.12, SE = 0.23, *p* < 0.05). No effect of condition was found for accuracy within the AMC or IWA group (AMC: biased = 77.8%, unbiased = 79.3%; IWA: biased = 60.6%, unbiased = 61.4%).

**Figure 3:**
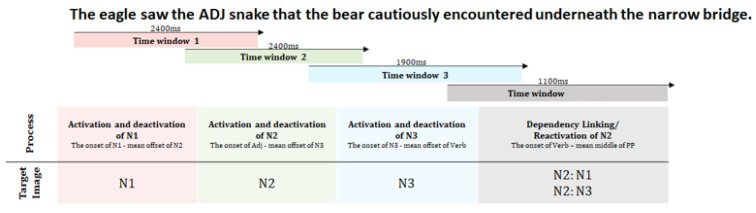
Specified windows of interest. The arrows represent when in the sentence a prespecified window starts and ends. The windows of analysis were overlapping as we wanted to capture the full morphology of the gaze pattern toward a targeted image. In these windows, we capture the activation (gazes toward) and deactivation (gazes away) parts of lexical processing. Here, we divided our sentence into four analysis windows to capture processing patterns via the gaze dynamics to the three images of the nouns that were mentioned in the sentence (here N1 represents the illustration of eagle, N2 the snake, N3 the bear).

*Online processing*: The subsequent analyses are focused on the condition differences between each group at specified windows of interest that are discussed in Figure 3. 

**Figure 4:**
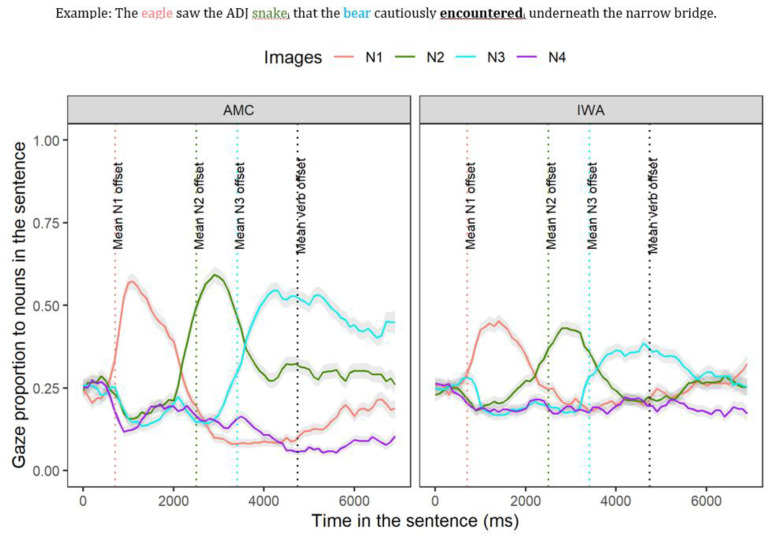
Mean gaze over time toward N1 (first noun, solid salmon line), N2 (second noun, solid green line), N3 (third noun, solid blue line), and a distractor image (solid purple line) averaged across conditions for each group which begins at the auditory onset of the sentence (N1), N4 (unrelated noun, purple line). Shaded areas represent 95% confidence intervals within subject. The dotted salmon line represents mean offset N1; the dotted green line represents mean offset N2; the dotted blue line represents mean offset N3; the dotted black line represents mean offset of the verb.

Figure 4 represents the time course of gazes during sentence processing for the two groups (AMC and IWA) across the two conditions (biased and unbiased). As shown in Figure 4 using colored dashed lines, there are critical parts of the sentences that were the focus of the analysis as described below.

### 3.1. Experimental Condition Effect on Lexical Processing

In an ongoing sentence, the way in which a word is processed will impact the processing of subsequent words in the sentence. Below, we present the processing patterns of nouns starting from the beginning of the sentence and moving forward in time in a linear fashion. In the following section, we examine the processing of each noun by analyzing their activation as well as de-activation patterns.

#### 3.1.1. Effect of Condition on Encoding the Noun Preceding the Manipulation (N1)

**Figure 5:**
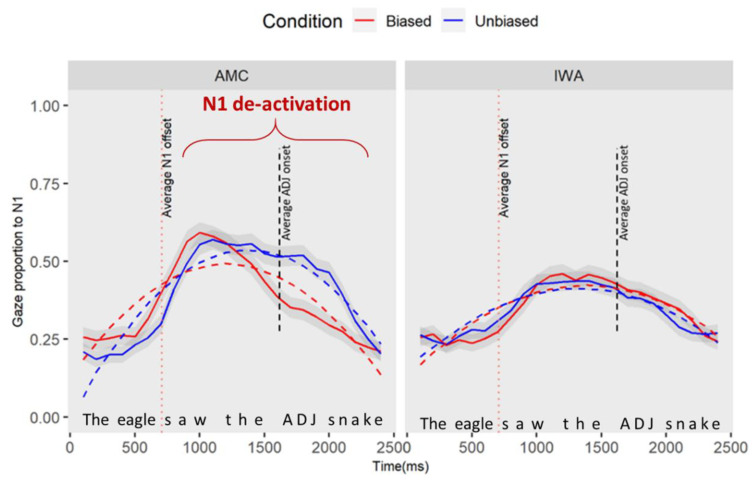
The plot captures the part of the sentence as “the eagle saw the/adjective/snake”. This plot demonstrates the gaze proportion differences to N1 between conditions and groups. Solid lines represent observed data and dashed lines represent the GCA model fit. The graphic representation of the model is showing the quadratic fit, yet the significant results for the condition effect were observed at the linear term.

Here, we examined if the adjective manipulation affected the processing of the preceding noun (e.g., deactivation of N1 upon hearing the adjective). We specified the window of analysis to occur 100 ms after the onset (this parameter allows time for planning and execution of an eye movement) of the first noun phrase until 2500 ms afterward (corresponding to the average offset of N2—“the eagle saw the/adjective/snake”, see Figure 3). Gaze data and curve fits for the interaction effects of group (AMC, IWA) and condition (biased and unbiased) for processing N1 are plotted in Figure 5 (see Appendix B for GCA modeling details). Upon visual inspection, unlike the IWA, the AMC show a stronger de-activation pattern in the biased condition compared to the unbiased condition.

The results of the individual parameter estimates revealed a simple effect of condition at the linear term which indicates that the average rate of change in gazes to and away from N1 for the AMC group changed in the biased condition compared to the unbiased condition (estimate = 0.31, SE = 0.13, *t* = 2.50, *p* = 0.02). The positive estimate indicated that the average rate of N1 processing (i.e., activation and deactivation over time) was lower in the unbiased condition compared to the biased condition. Moreover, there was an interaction effect of group and condition at the linear term (estimate = −0.37, SE = 0.14, *t* = −2.67, *p* = 0.01): the negative estimates on the linear terms indicated that the difference in the processing of N1 between conditions in the IWA group is smaller than the difference between the two conditions for the AMC group. Table 4 shows the full results of this analysis.

In summary, the GCA analysis suggests that there is a main effect of condition for the AMC group. To understand when in the time course the difference between conditions occurred, we conducted a permutation cluster analysis. In 2000 permuted samples, with an alpha of 0.05, the analyses revealed one significant cluster within 1640–2280 (cluster sum statistic = 97.03, *p* = 0.01) which corresponded with the onset of the adjective. Therefore, the difference between conditions for AMC individuals occurred at the point where the biased adjective was heard in the sentence. Moreover, when the adjective was semantically biased toward the next upcoming item (N2), AMC listeners showed an earlier disengagement from N1. 

#### 3.1.2. Effect of Condition on Encoding the Noun following the Manipulation (N2)

**Figure 6:**
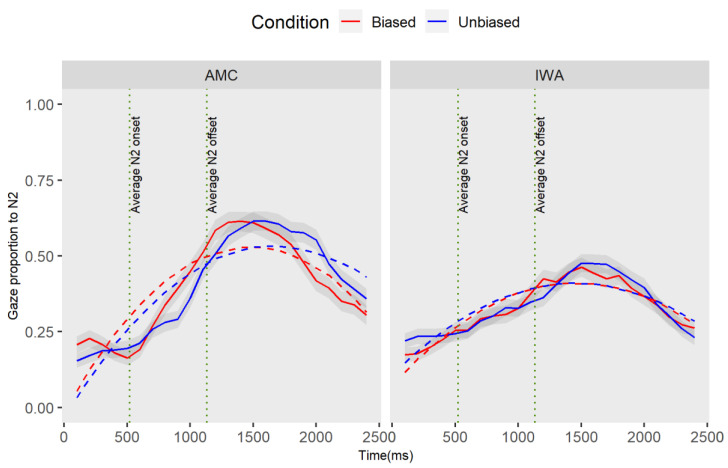
The plot captures the following part of the sentence “/adjective/snake that the bear”. This plot demonstrates the gaze proportion differences to N2 between conditions and groups. Solid lines represent observed data and dashed lines represent the GCA model fit. The graphic representation of the model is showing the quadratic fit, yet the marginal results for the group effect were observed at the intercept level, in addition to the interaction effect which was significant at the linear term.

Recall that time window 2 begins at the onset of the adjective until the average offset of noun 3 (“the/adjective/snake that the bear”, see Figure 3). Here, we seek to capture the effect of adjective bias on the processing patterns of the upcoming noun (N2). We employed the same analysis approach as described above in time window 1 (see Appendix C for GCA modeling details). Table 5 shows the full results of this analysis and Figure 6 shows the trajectory of effects.

The result of the individual parameter estimates revealed a marginal effect of the group at the intercept term (estimate = −0.05, SE = 0.02, *t* = −1.84, *p* = 0.066): this negative estimate, while not statistically significant at the 0.05 level, suggests that the average proportion of gazes toward N2 in the IWA is less than the AMC group. In addition, there was a marginal effect of condition for the AMC group at the linear term (estimate = 0.20, SE = 0.11, *t* =1.76, *p* = 0.08): the positive estimate indicates that that the average rate of N2 processing (i.e., activation and deactivation over time) was lower in the unbiased condition compared to the biased condition. While not statistically significant at the 0.05 level, this result suggests that the average rate of change in looking at the N2 for the AMC is different between the conditions. Furthermore, the results revealed a marginal interaction effect of group and condition at the linear term (estimate = −0.22, SE = 0.12, *t* = –1.78, *p* = 0.07): the negative estimates on the linear terms indicated that the difference in the processing of N2 between conditions in the IWA group is smaller than the difference between the two conditions in the AMC. Overall, the GCA analysis indicated no effect of the biased conditions for the IWA group. 

The GCA analysis revealed a marginal difference between the groups on the proportion of gazes toward N2. Moreover, the results revealed a marginal effect of condition in the AMC group. To determine when in the time course the difference between conditions occurred for the AMC group, we conducted a permutation cluster analysis. In the 2000 permuted samples, with an alpha of 0.05, the analyses revealed one significant cluster within 1920–2160 (cluster sum statistic = −40.56, *p* = 0.06) which corresponds with the offset of the N2. The marginal difference between conditions for AMC individuals occurred later when N3 was heard in the sentence. The biased condition resulted in an earlier disengagement from the N2 compared to the unbiased condition. Therefore, the condition difference is mainly reflected in the earliness of disengaging from the already activated item. The analysis of the downstream effect of adjectives on encoding the third noun (N3) is discussed in Appendix D.

In summary, the results of encoding the noun phrases (N1, N2, and N3) revealed that the presence of the adjective had a local effect during processing the first and second nouns in the sentence for the AMC group. In the biased condition, the AMC group revealed earlier disengagement from N1 and N2 upon hearing the next upcoming target nouns which means that the addition of adjective had facilitated the semantic integration processes as the speech stream unfold. However, IWA revealed impaired lexical processing patterns when compared with AMC. This was demonstrated by the lower rate of the magnitude of gaze proportions toward the targeted nouns upon hearing them in the sentence among IWA.

### 3.2. Experimental Condition Effect on Syntactic Retrieval 

Recall that in the post-verb window, successful dependency linking is evidenced as a re-activation of the direct-object noun (N2). The post-verb window is specified to begin at the onset of the verb until 1200 ms afterward (corresponding to “encountered_i_ underneath the narrow”, see Figure 3) to allow time for re-activation at the verb site as well as the spillover region. In this window, we explored whether re-activation occurred and inspected the presence of an interference effect during dependency linking by analyzing the gaze proportion of N2 (to-be-retrieved noun, henceforth “target”) relative to N1 and N3 (interfering nouns, henceforth “competitors”). Based on the cue-based parsing approach, upon encountering the verb, retrieval cues are triggered to search for a direct-object noun (N2); however, there are additional noun phrases whose features overlap with the target creating competition between the target (N2) and the non-target nouns (N1 and N3). Of importance is how the biased adjective is modulating the interference effects of non-target items across the groups. The evidence for the re-activation of N2 in the gap site (verb-frame) across the AMC and IWA groups is discussed in Appendix E. In the sections that follow, we examined each group separately and explored the effect of condition on the interference effects of N1 (3.2.1) and N3 (3.2.2) during re-activation of N2. 

#### 3.2.1. Effect of Condition on Re-Activation of N2 Relative to N1 at the Verb-Frame (Time Window 4b)

After establishing the presence of re-activation, the next question is whether the adjectives led to facilitation in retrieval. To understand the effect of condition on the proportion of N2 retrieval at the gap site, we built separate models for each group by including the interaction of fixed effect of images (N2 and N1) and condition. This interaction term reflects the extent to which the difference between N2 and N1 fixation time courses differed between conditions.

The individual parameter estimates for the AMC group (Table 6) revealed that the activation of N2 was lower in the unbiased condition compared to the biased condition (estimate = −0.031, SE = 0.02, *t* = −1.97, *p* < 0.05). 

The individual parameter estimates for IWA (Table 7) revealed that the activation level of the N1 competitor was higher in the unbiased condition compared to the biased one (estimate = 0.05, SE = 0.02, *t* = 2.15, *p* < 0.05). Moreover, the activation of target N2 at the intercept level was lower in the unbiased condition (estimate = −0.10, SE = 0.01, *t* = −6.26, *p* < 0.05) compared to the biased condition. There was a significant interaction effect at the linear term that revealed a later emerging increase in activation of N2 in the unbiased condition (estimate = 0.24, SE = 0.05, *t* = 4.79, *p* < 0.05). 

Overall, these results indicate a larger interference effect of N1 in the unbiased condition. Nevertheless, the lexical-semantic cues in the biased condition did not seem to benefit the IWA listeners enough to robustly reactivate N2 compared to N1. 

**Figure 7:**
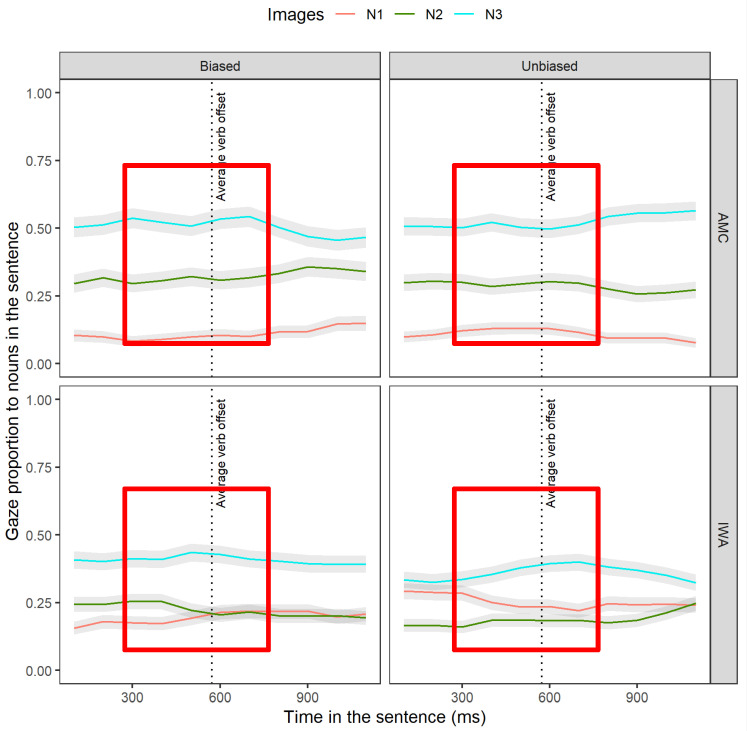
Averaged gaze proportions to N1, N2, and N3 between groups and conditions. This is the raw data observation of gaze toward N1, N2, and N3 in the verb-frame window. The gray shaded ribbons around the lines represent standard errors. The red boxes indicate the window in which the effect is expected.

Altogether, these sets of results from AMC and IWA indicated that the adjective type affected the dynamics of target re-activation at the gap site. In the biased condition, for the AMC group, the level of N2 re-activation was higher. Moreover, for the IWA group, the level of target N2 activation was higher while N1 interference was reduced (see Figure 7, red boxes). 

#### 3.2.2. Effect of Condition on Re-Activation of N2 Relative to N3 at the Verb-Frame (Time Window 4b)

After establishing the interference effect of N1, we conducted another analysis to observe the interference effect of N3 (subject of the relative-clause verb) during the re-activation of N2. Previously, we discussed that the recently activated representation of N3 can induce an interference effect during N2 re-activation. To investigate the effect of condition on the interference effect of N3, we repeated the same models for each group that was constructed before and looked at the interaction of condition with images N2 and N3. 

The results of the AMC group (Table 8) revealed that the re-activation of N2 was lower in the unbiased condition when compared to the biased one (linear term estimate = −0.10, SE = 0.05, *t* = −2.22, *p* < 0.05). Moreover, the activation of N3 was higher in the unbiased condition (intercept term estimate = 0.04, SE = 0.02, *t* = 2.02, *p* < 0.05) and its rate was increasing (linear term estimate = 0.21, SE = 0.06, *t* = 3.19, *p* < 0.05) when compared to the biased condition. These results revealed that the biased adjective affected the dynamics of target re-activation at the gap site and reduced the interference effect of N3. 

The results of the IWA group (Table 9) revealed an earlier increase in the rate of N2 activation overtime in the biased compared to the unbiased condition (linear term estimate 0.13, SE = 0.04, *t* = 3.09, *p* < 0.05). Yet, the activation dynamics of N3 did not change between the conditions. 

Altogether, these sets of results indicated that the biased adjective modulated the re-activation of N2 in both groups and reduced the interference effect of N3 in the AMC group (see Figure 7, red boxes). See Appendix F for the full summary of the results section. 

## 4. Discussion

In this study, we examined whether lexical-semantic cues as premodifiers of a target noun (N2) modulated the encoding (activation and deactivation gaze patterns) and retrieval (re-activation) dynamics of a noun phrase during the auditory processing of non-canonical sentences in both age-matched neurologically unimpaired listeners (AMC) and individuals with aphasia (IWA). We hypothesized that the lexical-semantic cues (in the form of adjectives) would facilitate (1) lexical encoding (i.e., boost representational access) and (2) downstream syntactic retrieval or dependency linking (i.e., increasing the probability of lexical retrieval at the gap site) during auditory sentence processing for both groups. The results revealed that the AMC group had a higher rate of activation and deactivation of nouns in the biased compared to the unbiased/neutral condition. Moreover, at the gap site, the accessibility of the target item (the displaced object noun, N2) was higher in the biased condition, which resulted in facilitated retrieval at the gap site. Our results from the AMC group are consistent with previous studies showing that semantically richer noun phrases that are encoded more ‘deeply’ are more accessible in memory at critical syntactic positions during sentence processing [24,25,32,33,34]. In contrast to the results found for the AMC group, the presence of biased adjectives did not affect the rate of lexical access of the target noun in the IWA group upon hearing the adjective in the sentence. Yet, in the post-verb-frame window, there was higher activation of target N2 and reduction in interference from the first noun competitor (N1). In the following sections, we discuss the results of the AMC group and then turn our discussion toward the IWA group to interpret the mechanism underlying the effect of the lexical-semantic cues (premodifier, adjective) during auditory sentence processing. 

### 4.1. Real-Time Dynamics of Lexical Encoding and Retrieval during Sentence Processing in Unimpaired Individuals 

In a self-paced reading paradigm, Hofmeister (2011) found that in neurotypical individuals, semantic complexity of the displaced object noun phrase or to-be-retrieved noun (e.g., “the injured and dangerous bear” versus “the bear”) resulted in longer reading times (i.e., longer encoding, or deeper processing) but then later yielded faster reading times at sentence-internal retrieval or re-activation sites [25]. The author suggested that richer representations containing typical or highly predictable feature combinations yielded retrieval facilitation at the retrieval site during sentence processing. In this study, using an eye-tracking-while-listening visual world paradigm, regarding the initial processing of the target noun, we found that the semantically biasing adjectives boosted the activation of representational features. We suggest that the presence of a biased adjective led to a greater spreading of activation such that accessing the set of features associated with the adjective primed the activation of semantic features of the target noun [60,61,62]. In other words, the semantically biased adjective increased the function of associative strengths and representational complexity during the processing of the target lexical item. Specifically, the presence of an adjectival cue as a premodifier provides a contextually unique feature for the target item that no other competitor shares. This can reduce the interference effect arising from the simultaneous presence of representations with overlapping features in memory (i.e., similarity-based interference) and improve the chances of its recoverability. As suggested by Nairne [35,63,64], the probability of retrieving a memory representation increases with the similarity or feature-overlap of the retrieval cues and target and decreases with the similarity of the cues to other memory candidates (see [65] for the full description of feature-based retrieval model of Nairne). Based on Nairne’s conceptual formulation, the probability of retrieving a representation *E*_1_, given a retrieval cue set *X*_1_, depends on the similarity or relatedness in features of *X*_1_ and *E*_1_, as well as the similarity or relatedness of *X*_1_ to other memory candidates (*E*_2_, *E*_3_, *E*_4_, …, *E*_n_). This ratio model is designed to describe the distinctiveness property of a cue (e.g., “venomous snake” versus “voracious snake” when both “bear” and “eagle” can be also voracious) during the retrieval.

(1) $$P_{r}\left(E_{1}|X_{1}\right)=\frac{S_{1}\left(X_{1},E_{1}\right)}{\sum S_{1}\left(X_{1},E_{n}\right)}$$

The numerator of this formulation refers to the similarity of *X*_1_ and *E*_1_, which varies as a function of the number of matching and mismatching features between the two terms which can be illustrated as the formulation below using the relating distance (*d*). This means that similar items (items containing few mismatching features) will be nearby items and produce the largest effects.

(2) $$s\left(X_{1},\;E_{1}\right)=e^{-d\left(E_{1},X_{1}\right)}$$

If the goal is to recover the representation *E*_1_ in the presence of a particular cue *X*_1_, the probability of retrieving *E*_1_ is highest when its features are similar to the cue *X*_1_ (the numerator of the equation), and dissimilar to other possible retrieval candidates (denominator). Therefore, the target retrieval is proportional to the cue-target match and inversely proportional to the amount of cue overload. Ultimately, the greater number of contextually unique features in *E*_1_, the greater it possesses a feature that no other competitor shares and, the greater the probability for compatibility with *X*_1_, and thus better chances for successful retrieval. In our case, the biasing adjectives make the target noun (snake: *E*_1_) distinct from the other competitor items (Eagle: *E*_2_ and bear: *E*_3_), thus reducing the level of cue overload, which can result in a higher probability of target item *E*_1_ retrieval. Moreover, using this feature-based model of retrieval, Hoffmeister et al. (2013) suggested that increasing representational complexity increases the probability that some features will be unique and therefore helps distinguish a representation from other competitors in memory [65]. With respect to the cue-based retrieval theories, such uniqueness may create a better match with the set of retrieval cues at the gap site [9]. Therefore, adding unique information can be quite helpful for memory retrieval. In the present study, regarding the downstream effects, the additional lexical-semantic information provided by the biasing adjective increases the representational complexity of the target noun and increases the distinctiveness of the target at the time of retrieval for neurotypical individuals. The results revealed that the AMC group disengaged from the first noun phrase earlier upon hearing the biasing adjective noun phrase when compared to the neutral adjective in the unbiased condition. Moreover, they retrieved (reactivated) the target N2 earlier in the retrieval site (post-verb-frame window) and manifested an increase in its rate of re-activation in the biased condition compared to the unbiased condition. Therefore, the results from AMC are consistent with previous studies showing that semantically richer nouns are more accessible in memory [24,25,32,34].

### 4.2. Real-Time Dynamics of Lexical Encoding and Retrieval during Sentence Processing in Individuals with Aphasia 

Unlike the AMC group, IWA did not demonstrate sensitivity to the lexical-semantic cues (biased adjectives) in their rate of initial lexical access. However, their re-activation processes of the displaced item changed in the post-verb-frame window. IWA demonstrated a reduction in interference-effect arising from the competitor item in the sentence. The lack of sensitivity of IWA to the lexical-semantic cue during real-time processing could be attributed to their inefficiency in accessing or maintaining the representational features in real time. Research exploring real-time processing in aphasia has suggested that these individuals have a delay in lexical access causing the critical semantic features to be unavailable for fast-acting syntactic processes [13,16,37]. Yet, the deficits could be overcome when the rate of auditory input is slowed down and the time constraints for retrieval are relaxed [16]. This is in line with studies with neurotypical individuals that have suggested that the addition of time at specific points during processing allows for a deeper encoding of sentential constituents, leading to a strengthened representation [52,66]. In the current study, we aimed to strengthen the representations via biasing adjectives, though the approach was unsuccessful. One explanation for the lack of sensitivity of IWA to the contextual cue could be attributed to the interference of active representations that are outside of the scope of other sentential element constraints such as phonological form [67,68,69]. Although we do not have direct evidence to support this, it has been suggested that impairments in cognitive control processes (such as cognitive flexibility and inhibitory control) can increase the interference from context-independent distractors during sentence processing [70]. Investigations into the effect of these impaired processes in IWA should be considered in future endeavors.

### 4.3. The Underlying Nature of Lexical-Semantic Processing Deficit in Aphasia

Nozari (2019) introduced a theoretical framework that explains all the empirical findings surrounding lexical-access deficits in aphasia [71]. The framework, which is based on language production, can be generalized to comprehension processes as it pertains to shared mechanisms (namely representational semantic storage and cognitive control processes) that are involved in both production and comprehension. Nozari (2019) demonstrated that lexical access deficits in aphasia can have two distinct etiologies by presenting a case of a double dissociation between two IWA. One case showed a profile of impaired activation of semantic features of the target lexical items (activation deficit), while the other case showed a profile compatible with impaired inhibition of competing for lexical items (inhibition deficit). Those IWA with activation deficits suffer from lower-than-normal activation of representational features (semantic or phonological) which can lead to smaller differences between items during the spread of activation and ultimately impede the absolute selection of an item [72]. Those IWA with inhibition deficits suffer from increased activation of semantic competitors which hinges on the malfunction of the inhibitory process that suppresses the activation of unrelated representations. Deficits in any of these mechanisms can explain why IWA, upon first hearing a target noun, demonstrated a lack of sensitivity to the distinctiveness in the biased condition. This framework is related to the feature-based retrieval formulation of Nairne (2006 and references therein) which expresses that the probability that a target representation will be selected depends on cue-target features match and distinctiveness of the target from competitor items which dictate the level of interference within the potential representations. Our results demonstrate that IWA do not appear to be initially sensitive to the distinctiveness property of a cue during the fast-acting real-time processing of sentences as they have impairments in the timely processing of these functions. However, since they evinced a later emerging reduced interference effect between the target noun and the competitor noun (N1) during the post-verb-frame window, this suggests that distinctiveness is processed, just delayed.

Altogether, if the intention is to mitigate initial delay in lexical access, then the addition of biasing adjectives as premodifiers may not be an ideal approach for IWA to boost representational access in the memory as they have a delay in timely access to representational features. Future investigations may explore adding modifiers after the noun (post-modifiers, e.g., “It was the bear with large claws that the hunter chased into the evening”, as compared to a matched sentence with a neutral preposition phrase after the target noun) as those may offer a better route for enriching the semantic features of a representation. Post-modifiers may be more efficiently encoded by IWA during online sentence processing. It is suggested from neurotypical studies that in the case of postmodifiers, the memory representation (semantic and syntactic features) of the head noun becomes reactivated as the modifying information is being encoded [11]. Since the full lexical semantics of the head noun is available in the case of postmodifiers, an immediate re-activation of both syntactic and semantic information can lead to more robust representational access and allows time for lexical processing to be fully executed. Future studies need to look at the effect of pre and postmodifiers in sentence processing patterns of individuals with aphasia. Another viable approach to modulate sentence processing and reduce the interference effect for IWA is to directly manipulate the representational features of the target item and make them inherently mismatching from other competitor items in the sentence. In a similar vein, previous reading studies with neurotypical individuals have shown that a mismatch in the properties of encoded referents of the sentence, such as “the general” and “Christopher” in (5b) can minimize the similarity-based interference effects when compared to (5a) and therefore increase the probability of on-time target retrieval [5,6,73]. This approach could be more useful for IWA rather than increasing the syntactic and semantic representational complexity of the to-be-retrieved item by adding modifiers.

(5a) It was *the general*_i_ that the lawyer chased_i_ <the general> in the office yesterday.
(5b) It was *the general*_i_ that Christopher chased_i_ <the general> in the office yesterday.
(5c) It was the *victorious four-star general_i_* that the lawyer chased_i_ <the general> in the office yesterday.

One limitation in this study is the small sample size of the aphasia group. The sample size limited the ability to conduct individual-level analyses. Moving forward, it would be useful to relate features of stroke-induced lesions, such as size, location, and white-matter damage, to variability in language outcomes across individuals with aphasia.

## 5. Conclusions

Altogether, the current study improves our understanding of how words are encoded, processed against competitor items, and retrieved during language comprehension in neurologically unimpaired as well as impaired populations. Here, we demonstrate that a boost in representational access via premodifiers (biasing adjectives) can facilitate the syntactic processing of unimpaired populations. However, disruption in the timely activation of compatible representations can reduce the sensitivity to premodifying lexical-semantic cues among IWA.

## Figures and Tables

**Table 1:** Gaze movement metrics of specific sentence-level processes.

Processing Level	Gaze Movement Pattern
Lexical access	Gaze movement toward a visual representation of a target noun upon hearing it in the sentence
Lexical integration	After lexical access, gaze divergence from a previously accessed target noun indicates its integration into the syntactic structure
Dependency linking	Gaze movement that returns to a noun representation that was previously activated (re-activation) when it is syntactically licensed
Interference effects	An equivalent proportion of gazes toward related as well as non-target nouns (i.e., that are not relevant at a given point in a sentence) indicates an individual’s susceptibility to the interference effect

**Table 2:** IWA Participants’ characteristics (*n* = 11).

IWA	Sex	Years Post-Stroke	Age at Testing	Years of Education	Aphasia Subtype	Lesion Location	BDAE-v3	WAB-R AQ	SOAP-SR (%)	SOAP- OR (%)
**009**	M	15	55	17	Mixed non-fluent	Large L lesion, IFG (BA 44/BA45) w/posterior	4	67.7	60%	40
**017**	M	18	66	15	Anomic	L anterior cerebral and middle cerebral infarct	4	95.4	100	90
**101**	M	9	67	20	Broca	Large L lesion posterior IFG (BA 44) w/posterior	2	82.6	100	30
**130**	M	8	63	16	Broca /Anomia	L IPL with posterior ext. sparing STG	4	90.5	75	55
**140**	F	16	42	-	-	L MCA infarct	2	75.7	80	30
**151**	F	7	65	16	Anomic	L MCA infarct with subcortical extension	4	95.8	100	100
**159**	F	6	64	16	Broca	L MCA infarct	3	92.4	100	70
**165**	F	4	64	12	Broca	L MCA infarct	3	ND	80	60
**169**	M	4	59	12	Broca	L MCA infarct	2	28.2	80	40
**190**	F	6	76	12	Broca	Left superior temporal lobe	3	88.2	90	40
**191**	M	1	57	16	Broca	L MCA infarct	4.5	98.4	100	60
**AMC Group**	Ages 57–66 years (mean = ~61.9); 7 females, 4 males; education 14–18 years (mean = 15.7) *									

M = male, F = female; L = left; LH = left hemisphere; BA = Brodmann area; IPL = inferior parietal lobule; STG = superior temporal gyrus; MCA = middle cerebral artery. BDAE = Boston Diagnostic Aphasia Examination (0 = no usable speech or auditory comprehension; 5 = minimal discernable speech handicap). SOAP SR = average percent correct on subject-relative items from the SOAP Test of Auditory Sentence Comprehension. SOAP OR = average percent correct of object relative items from the SOAP Test of Auditory Sentence Comprehension. * Missing education data for four AMC individuals.

**Table 3:** Example of experimental sentence and visual stimuli.

Condition	Sample Sentence	Visual Array
Unbiased Adjective	“The eagle saw the voracious snake that the bear cautiously encountered underneath the narrow bridge.”	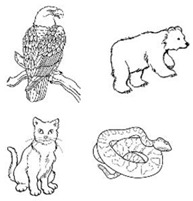
Biased Adjective	“The eagle saw the venomous snake that the bear cautiously encountered underneath the narrow bridge.”	

**Table 4:** Results of GCA analysis for time window 1 (processing N1).

Predictors	Estimates	CI	P (Two Tailed)
(Intercept)	0.37	0.31–0.42	<0.001
Linear	−0.08	−0.31–0.15	0.506
Quadratic	−0.53	−0.72–−0.34	<0.001
Condition [Unbiased]	0.02	−0.03–0.07	0.497
Group [IWA]	−0.02	−0.09–0.05	0.512
**Linear × Condition [Unbiased]**	**0.31**	**0.07–0.56**	**0.012**
Quadratic × Condition [Unbiased]	−0.05	−0.24–0.14	0.589
Linear × Group [IWA]	0.19	−0.09–0.47	0.178
Quadratic × Group [IWA]	0.20	−0.03–0.43	0.093
Condition [Unbiased] × Group [IWA]	−0.02	−0.06 0.02	0.367
**(Linear × Condition [Unbiased]) × Group [IWA]**	**−0.37**	**−0.64–−0.10**	**0.008**
(Quadratic × Condition [Unbiased]) × Group [IWA]	0.07	−0.14–0.27	0.523

Note: The table provides the test of the full model including the interaction of group and condition on the intercept, linear, and quadratic time terms. The AMC group and the biased condition are set as the reference estimates. Results in **boldface** are presented in the text.

**Table 5:** Results of GCA analysis for time window 2 (processing N2).

Predictors	Estimates	CI	P (Two Tailed)
(Intercept)	0.38	0.33–0.43	<0.001
Linear	0.35	0.07–0.62	0.014
Quadratic	−0.51	−0.71–−0.31	<0.001
Condition [Unbiased]	0.01	−0.05–0.07	0.763
**Group [IWA]**	**−0.05**	**−0.11–0.00**	**0.066**
**Linear × Condition [Unbiased]**	**0.20**	**−0.02–0.41**	**0.078**
Quadratic × Condition [Unbiased]	0.11	−0.07–0.30	0.237
Linear × Group [IWA]	−0.12	−0.48–0.23	0.497
Quadratic × Group [IWA]	0.18	−0.06–0.43	0.149
Condition [Unbiased] × Group [IWA]	−0.00	−0.06–0.06	0.971
**(Linear × Condition [Unbiased]) × Group [IWA]**	**−0.22**	**−0.46–0.02**	**0.075**
(Quadratic × Condition [Unbiased]) × Group [IWA]	−0.08	−0.27–0.10	0.362

Note: The table provides the test of the full model including the interaction of group and condition on the intercept, linear, and quadratic time terms. The AMC group and the biased condition are set as the reference estimates. Results in **boldface** are presented in the text.

**Table 6:** Results of GCA analysis of AMC data for time window 4 (processing N2 relative to N1).

Predictors	Estimates	CI	P (Two Tailed)
(Intercept)	0.12	0.04–0.19	0.002
Linear	0.01	−0.12–0.14	0.857
Quadratic	0.03	−0.04–0.10	0.413
Images [N2]	0.19	0.11–0.27	<0.001
Condition [Unbiased]	0.00	−0.04–0.05	0.924
Linear × Images [N2]	0.05	−0.11–0.20	0.547
Quadratic × Images [N2]	−0.02	−0.12–0.08	0.725
Linear × Condition [Unbiased]	−0.06	−0.17–0.06	0.333
Quadratic × Condition [Unbiased]	−0.07	−0.15–0.00	0.059
**Images [N2] × Condition [Unbiased]**	**−0.03**	**−0.06–−0.00**	**0.049**
(Linear × Images [N2]) × Condition [Unbiased]	−0.04	−0.14–0.07	0.473
(Quadratic × Images [N2]) × Condition [Unbiased]	0.05	−0.05–0.16	0.323

Note: The table provides the test of the full model including the interaction of condition and images of interest (N1 and N2) on the intercept, linear, and quadratic time terms. The biased condition and the N1 are set as the reference estimate. Results in **boldface** are presented in the text.

**Table 7:** Results of GCA analysis of IWA data for time window 4 (processing N2 relative to N1).

Predictors	Estimates	CI	P (Two Tailed)
(Intercept)	0.20	0.14–0.25	<0.001
Linear	0.06	−0.01–0.12	0.081
Quadratic	−0.03	−0.08–0.02	0.255
Images [N2]	0.03	−0.02–0.09	0.217
**Condition [Unbiased]**	**0.05**	**0.00–0.10**	**0.031**
Linear × Images [N2]	−0.12	−0.21–−0.04	0.003
Linear × Images [N2]	0.03	−0.04–0.10	0.362
**Linear × Condition [Unbiased]**	**−0.11**	**−0.19–−0.04**	**0.004**
Quadratic × Condition [Unbiased]	0.07	0.00–0.15	0.044
**Images [N2] × Condition [Unbiased]**	**−0.10**	**−0.13–−0.07**	**<0.001**
**(Linear × Images [N2] × Condition [Unbiased]**	**0.24**	**0.14–0.34**	**<0.001**
(Quadratic × Images [N2] × Condition [Unbiased]	−0.05	−0.15–0.05	0.359

Note: The table provides the test of the full model including the interaction of condition and images of interest (N1 and N2) on the intercept, linear, and quadratic time terms. The biased condition and the N1 are set as the reference estimate. Results in boldface are presented in the text.

**Table 8:** Results of GCA analysis of AMC data for time window 4 (processing N2 relative to N3).

Predictors	Estimates	CI	P (Two Tailed)
(Intercept)	0.31	0.21–0.41	<0.001
Linear	0.07	−0.08–0.21	0.368
Quadratic	0.01	−0.06–0.08	0.777
Images [N3]	0.21	0.07–0.35	0.003
Condition [Unbiased]	−0.03	−0.07–0.00	0.080
Linear × Images [N3]	−0.10	−0.31–0.11	0.347
Quadratic × Images [N3]	−0.07	−0.17–0.03	0.168
**Linear × Condition [Unbiased]**	**−0.10**	**−0.19–−0.01**	**0.026**
Quadratic × Condition [Unbiased]	−0.02	−0.11–0.07	0.654
**Images [N3] × Condition [Unbiased]**	**0.04**	**0.00–0.08**	**0.044**
**(Linear × Images [N3]) × Condition [Unbiased]**	**0.21**	**0.08–0.33**	**0.001**
(Quadratic × Images [N3]) × Condition [Unbiased]	0.11	−0.02–0.23	0.089

Note: The table provides the test of the full model including the interaction of condition and images of interest (N2 and N3) on the intercept, linear, and quadratic time terms. The biased condition and the N2 are set as the reference estimate. Results in **boldface** are presented in the text.

**Table 9:** Results of GCA analysis of IWA data for time window 4 (processing N2 relative to N3).

Predictors	Estimates	CI	P (Two Tailed)
(Intercept)	0.23	0.15–0.31	<0.001
Linear	−0.07	−0.14–0.01	0.073
Quadratic	0.00	−0.07–0.07	0.986
Images [N3]	0.18	0.07–0.28	0.001
Condition [Unbiased]	−0.04	−0.09–0.01	0.118
Linear × Images [N3]	0.04	−0.05–0.14	0.368
Quadratic × Images [N3]	−0.02	−0.12–0.07	0.638
**Linear × Condition [Unbiased]**	**0.13**	**0.05–0.21**	**0.002**
Quadratic × Condition [Unbiased]	0.03	−0.05–0.10	0.474
Images [N3] × Condition [Unbiased]	0.01	−0.03–0.04	0.687
(Linear × Images [N3]) × Condition [Unbiased]	−0.09	−0.19–0.02	0.101
(Quadratic × Images [N3]) × Condition Unbiased]	−0.08	−0.19–0.02	0.128

Note: The table provides the test of the full model including the interaction of condition and images of interest (N2 and N3) on the intercept, linear, and quadratic time terms. The biased condition and the N2 are set as the reference estimate. Results in **boldface** are presented in the text.

**Figure A1:**
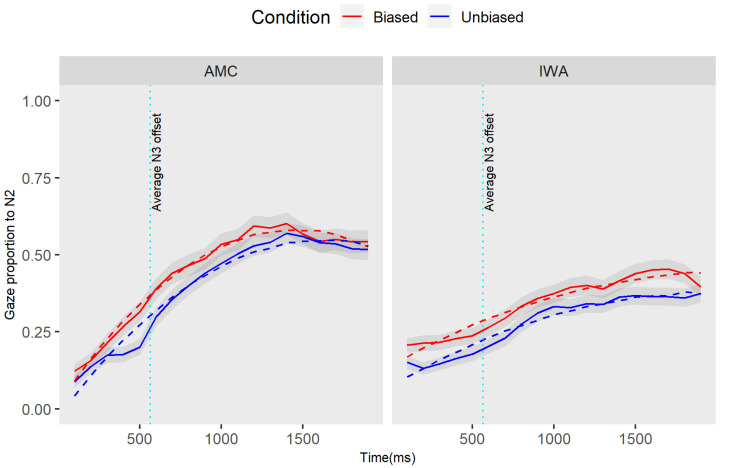
Gaze proportion differences toward N3 between conditions and groups. Solid lines represent observed data and dashed lines represent the GCA model fit.

**Figure A2:**
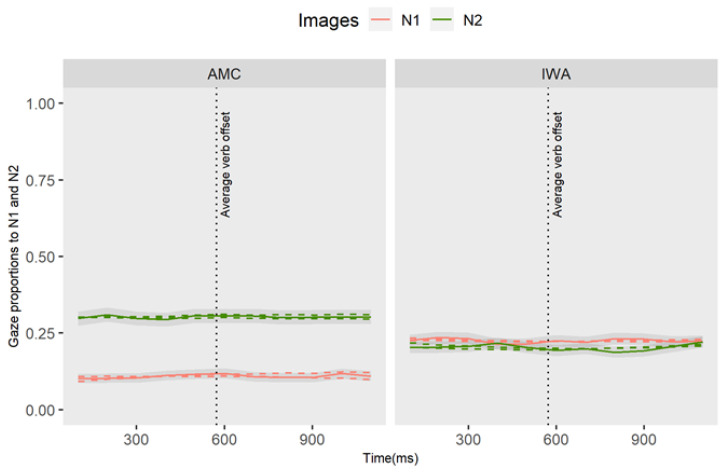
Gaze proportion differences to N1 and N2 between groups. Solid lines represent observed data and dashed lines represent the GCA model fit.

## Data Availability

The data used to support the findings of this publication can be requested from the corresponding author upon request.

## Appendix A

**Table A1:** The list of sentence stimuli used in this study is shown in the table below.

Unbiased Adjective	Biased Adjective
The duck followed the perfect kitten that the cow deliberately nudged across the grassy meadow.	The duck followed the playful kitten that the cow deliberately nudged across the grassy meadow.
The veterinarian greeted the popular king that the criminal mistakenly expected at the stunningly lavish gala.	The veterinarian greeted the powerful king that the criminal mistakenly expected at the stunningly lavish gala.
The scorpion annoyed the anxious bull that the bee constantly pestered in the abandoned railroad yard.	The scorpion annoyed the angry bull that the bee constantly pestered in the abandoned railroad yard.
The crocodile spied the weird owl that the chameleon momentarily faced in the exotic animal show.	The crocodile spied the wise owl that the chameleon momentarily faced during the exotic animal show.
The crab helped the coy puppy that the rabbit relentlessly teased before playful tussle.	The crab helped the cute puppy that the rabbit relentlessly teased before the playful tussle.
The lawyer visited the forgetful gymnast that the butler allegedly helped with the illegal cover-up.	The lawyer visited the flexible gymnast that the butler allegedly helped with the illegal cover-up.
The magician passed the redheaded nun that the mailman compassionately soothed after the traumatic event.	The magician passed the religious nun that the mailman compassionately soothed after the traumatic event.
The ladybug observed the smelly bat that the opossum deliberately avoided near the historic monument.	The ladybug observed the scary bat that the opossum deliberately avoided near the historic monument.
The astronaut approached the sad jockey that the salesman incorrectly judged throughout the dinner party.	The astronaut approached the short jockey that the salesman incorrectly judged throughout the dinner party.
The otter spotted the shiny octopus that seagull unsurprisingly smelled after the hot and sunny day.	The otter spotted the slimy octopus that seagull unsurprisingly smelled after the hot and sunny day.
The deer noticed the male gorilla that the hummingbird thoroughly amused with the acrobatic display.	The deer noticed the mean gorilla that the hummingbird thoroughly amused with the acrobatic display.
The ostrich recognized the delightful toucan that the baboon hesitantly touched during the bizarre encounter.	The ostrich recognized the colorful toucan that the baboon hesitantly touched during the bizarre encounter.
The spider scared the live rooster that the porcupine accidentally bumped on the side of the country road.	The spider scared the loud rooster that the porcupine accidentally bumped on the side of the country road.
The dentist helped the tired maid that the plumber heartlessly cheated in spite of the cautious investment.	The dentist helped the tidy maid that the plumber heartlessly cheated in spite of the cautious investment.
The orangutan examined the defenseless cockroach that the parrot quickly located near the bottom of the staircase.	The orangutan examined the disgusting cockroach that the parrot quickly located near the bottom of the staircase.

## Appendix B. Model Details for Analyzing the Noun Preceding Adjective

The data for this analysis was composed of gaze proportions to N1 across the window for the 9 AMC and 10 IWA. This window captures the full dynamics of N1 processing, which includes its activation and deactivation pattern over time as the speech unfolds. We began the GCA analysis using a base model that included only the time terms (linear and quadratic) without any modulation of group and condition. The addition of group (AMC vs. IWA) and condition (biased vs. unbiased) parameters to the model significantly improved the model fit (χ^2^(12) = 154.91, *p* < 0.001). Of interest was the interaction term between the condition and group reflects the extent to which the difference in N1 gaze proportion between biased and unbiased conditions differed between participant groups. The AMC group served as the comparison group and coefficients were estimated for the IWA relative to the AMC group.

## Appendix C. Model Details for Analyzing the Noun following Adjective

To capture the full pattern of N2 processing (i.e., its activation and deactivation over time) and examine the effect of the biased adjective, we specified the window of analyses to include the onset of adjective until 2500 ms afterward (corresponding to the average offset of N3—“the/adjective/snake that the bear”). The data for this analysis was composed of N2 gaze proportion over time for the 9 AMC and 10 IWA participants. Here, we built a baseline model including only the time terms (linear and quadratic). The addition of group (AMC vs. IWA) and condition (biased vs. unbiased) improved the model fit (χ^2^(12) = 161.43, *p* < 0.001). This interaction term reflects the extent to which N2 gaze proportion differences between biased and unbiased conditions differed between participant groups.

## Appendix D. Downstream Effect of Condition on Encoding the Noun after the Manipulation (N3)

For this analysis, the window was specified at the onset of N3 until 2000 ms afterward (corresponding to the average offset of the verb—“the bear cautiously encountered”). We began the GCA analysis using the base model that includes time without any modulation of group and condition. The addition of group (AMC vs. IWA) and condition (biased vs. unbiased) improved the model fit (χ^2^(12) = 150.41, *p* < 0.001). See Figure A1 for the gaze data and curve fits for this interaction model.

The result of the individual parameter estimates (Table A2) revealed a marginal effect of condition for N3 processing in the AMC group at the intercept term (estimate = −0.06, SE = 0.03, *t* = −1.09, *p* = 0.06): this negative estimate indicated that the average proportion of gazes toward N3 in the unbiased condition was less than the biased condition. Furthermore, there was an effect of the group at the intercept term (estimate = −0.11, SE = 0.05, *t* = −2.09, *p* = 0.04), which indicated that the average proportion of gazes toward N3 for IWA was less than the AMC group (this result corresponds to the biased condition which is the reference estimate). Additionally, the main effect of the group was also significant at the quadratic term (estimate = 0.23, SE = 0.10, *t* = 2.22, *p* = 0.03), which is indicative of a steeper rise and fall (curvature inflection point) for the AMC compared to IWA in the biased condition.

**Table A2:** Results of GCA analysis for time window 3.

Predictors	Estimates	CI	P (Two Tailed)
(Intercept)	0.44	0.36–0.53	<0.001
Linear	0.53	0.26–0.81	<0.001
Quadratic	−0.31	−0.47–−0.15	<0.001
**Condition [Unbiased]**	**−0.06**	**−0.12–0.00**	**0.056**
**Group [IWA]**	**−0.11**	**−0.22–−0.01**	**0.036**
Linear × Condition [Unbiased]	0.09	−0.14–0.32	0.458
Quadratic × Condition [Unbiased]	0.07	−0.09–0.22	0.387
Linear × Group [IWA]	−0.20	−0.56–0.16	0.275
**Quadratic × Group [IWA]**	**0.23**	**0.03–0.43**	**0.027**
Condition [Unbiased] × Group [IWA]	0.01	−0.06–0.07	0.853
(Linear × Condition [Unbiased]) × Group [IWA]	−0.04	−0.32–0.24	0.774
(Quadratic × Condition [Unbiased]) × Group [IWA]	−0.08	−0.26–0.10	0.397

Note: The table provides the test of the full model including the interaction of group and condition on the intercept, linear, and quadratic time terms. The AMC group and the biased condition are set as the reference estimates. Results in **boldface** are presented in the text.

The marginal effect of the condition which was found in the AMC group was reevaluated using the permutation cluster analysis (2000 permuted samples, with an alpha of 0.05). The analysis did not result in any significant clusters that would indicate differences between conditions. The cluster analysis did not validate the GCA finding, and therefore we cannot report any consistent effect of group or condition difference for N3 processing.

## Appendix E. Evidence of N2 Re-Activation at the Verb-Frame (Time Window 4)

We inspected the re-activation of N2 relative to N1 as an index of syntactic re-activation at the gap site window, for two reasons: first, if gaze proportions to N2 and N1 were similar, then it means that the listeners must have maintained activation for items on the screen regardless of their syntactic roles. In other words, at this verb-frame position, individuals’ gazes must be away from N1 as the syntactic role of these items should have been already assigned. The activation of N1 at this verb-frame position would only indicate the presence of an interference effect. However, at this point in the sentence, there are two NPs that have not yet been fully integrated into the syntactic structure; that is N2 and N3. It is to be expected that the most recently encountered N3 would have high gazes as its traces of representation can remain active after hearing the verb. Therefore, if syntactic linking is triggered at the verb offset, then we would be expecting higher gazes toward N2 and not N1. To indicate if individuals in each group have shown evidence of re-activation at the verb-frame, we formed a second-order orthogonal polynomial and added the fixed effect of group (IWA vs. AMC) and gazes toward the images of interest (N2 vs. N1) to the model. See Figure A2 for the gaze data and curve fits for this interaction model.

Adding the group and images and their interaction with the higher-order time terms improved the baseline model fit (χ^2^(12) = 697.09, *p* < 0.001). This interaction term reflects the extent to which the difference between N2 (target) and N1 (competitor) gaze time courses differed between participant groups. In Table A3 we report the results of individual parameter estimates of the full quadratic model. The analysis revealed an effect of the group such that the average gaze of IWA toward the competitor image (N1) was higher than the AMC group (estimate = 0.10, *p* < 0.05). There was also an interaction effect showing that the IWA group had a significantly lower intercept term (estimate = −0.19, SE = 0.05, *t* = −3.88, *p* < 0.001) relative to the AMC group. The effect on the intercept term indicates that the difference in overall fixation of N1 vs. N2 was smaller for the IWA group than for the AMC group. As shown in the plots, the AMC group did not maintain activation of N1 and had a higher proportion of gazes toward N2, which is indicative of reduced interference effect in this group.

Altogether, we can summarize that IWA were experiencing interference effects during the verb-frame window (i.e., gap site where syntactic dependency linking must occur). The next analysis investigates whether the condition modulated the pattern of gazes toward N2 at the verb-frame position.

**Table A3:** Results of GCA analysis for time window 4.

**Predictors**	**Estimates**	**CI**	**P (Two Tailed)**
(Intercept)	0.12	0.06–0.18	<0.001
Linear	−0.01	−0.10–0.07	0.793
Quadratic	−0.01	−0.05–0.03	0.654
Images [N2]	0.17	0.10–0.24	<0.001
**Group [IWA]**	**0.10**	**0.02–0.18**	**0.016**
Linear × Images [N2]	0.02	−0.09–0.14	0.696
Quadratic × Images [N2]	0.01	−0.05–0.07	0.764
Linear × Group [IWA]	0.01	−0.10–0.13	0.832
Quadratic × Group [IWA]	0.02	−0.04–0.08	0.532
**Images [N2]** × **Group [IWA]**	**−0.19**	**−0.29–−0.09**	**<0.001**
(Linear × Images [N2]) × Group [IWA]	−0.02	−0.18–0.14	0.803
(Quadratic × Images [N2]) × Group [IWA]	−0.00	−0.08–0.08	0.982

Note: The table provides the test of the full model including the interaction of group and images of interest (N1 and N2) on the intercept, linear, and quadratic time terms. The AMC group and the N1 are set as reference estimates. Results in boldface are presented in the text.

## Appendix F. Summary of Results

**Table A4:** Summary of the results for the online sentence processing of AMC and IWA based on GCA and Cluster analyses.

**Process**				
	**Activation and Deactivation of N1**	**Activation and Deactivation of N2**	**Activation and Deactivation of N3**	**Dependency Linking/Re-Activation of N2**
**Results-AMC**	Across the entire time course of processing N1, there was an effect of condition. The effect emerges upon hearing the adjective. In the biased condition, deactivation (looking away from N1 when processing the adjective occurred earlier than in the unbiased condition.	Across the entire time course of processing N2, there was a marginal effect of condition. The difference emerges at the offset of N2. In the biased condition, N2 was deactivated earlier as compared to the unbiased condition.	AMC did not show a significant effect of condition for activation and deactivation of N3.	There was a condition effect for the AMC group such that they revealed a higher rate of activation of N2 in the biased compared to the unbiased condition. Moreover, the level and rate of activation of N3 were lower in the biased condition.
**Results-IWA**	IWA did not show an effect of condition for N1 processing.	IWA did not show an effect of condition for N2.	IWA did not show an effect of condition for N3.	IWA showed an earlier rise in the re-activation of N2 in the biased condition They revealed a reduced interference effect in the biased condition as manifested by reduced looks to N1. They revealed no different gaze patterns toward N3 between the conditions.
